# Epidemia (*romé*) de sarampión entre los Hitnü (Arauca, Colombia, 1964): una historia basada en etnografía

**DOI:** 10.1590/0102-311XES116124

**Published:** 2025-07-04

**Authors:** Claudia Amaya-Castellanos, Antonio Lobo-Guerrero, Esther Jean Langdon, Álvaro J. Idrovo, Francisco Ortega

**Affiliations:** 1 Universidad Industrial de Santander, Bucaramanga, Colombia.; 2 Universitat Rovira i Virgili, Tarragona, España.; 3 Fundación Etnollano, Bogotá, Colombia.; 4 Universidade Federal de Santa Catarina, Florianópolis, Brasil.; 5 Instituciò Catalana de Recerca i Estudis Avancats, Barcelona, España.

**Keywords:** Epidemia, Sarampión, Salud de Poblaciones Indígenas, Etnografía, Epidemic, Measles, Health of Indigenous Peoples, Ethnography, Epidemia, Sarampo, Saúde de Populações Indígenas, Etnografia

## Abstract

Este trabajo describe la epidemia de sarampión de 1964 entre el pueblo Hitnü de Arauca, Colombia, desde la perspectiva de los propios indígenas. Para ello se realizó un estudio de Antropología Histórica usando diarios de campo de dos abordajes etnográficos (1976-1982 y 2023-2024). Los datos recolectados hace casi 50 años, fueron de los sobrevivientes de la epidemia, y los registros más recientes fueron de indígenas Hitnü mayores, que conocieron los hechos por tradición oral de sus padres. Los resultados muestran que el pueblo Hitnü reconoce las características de lo que constituye una epidemia (*romé*) de sarampión. Además, se plantean elementos de carácter simbólico que acompañan las explicaciones sobre la epidemia, y se postula que sus interpretaciones fueron influenciadas por el contexto histórico y sociopolítico del momento, caracterizado por luchas entre indígenas y no indígenas. Esta experiencia muestra el valor de la Etnografía para reconstruir hechos relacionados con epidemias y explorar las emergencias sanitarias desde la perspectiva de los afectados y relatadas por etnógrafos, lejos de las frecuentes narrativas hegemónicas, que refuerzan las desigualdades, deslegitimando o ignorando no sólo los conocimientos y capacidades resolutivas de los indígenas respecto a este tipo de eventos, sino sus interpretaciones, las cuales como en el caso de los Hitnü y otros grupos de Sudamérica, están vinculados con el relacionamiento que han establecido con los no indígenas a través del tiempo.

## Abstract (en)

This study describes the 1964 measles epidemic among the Hitnü people of Arauca, Colombia, based on the perspective of this Indigenous people. For this, a Historical Anthropology study was carried out with field diaries based on two ethnographic approaches (1976-1982 and 2023-2024). The data were collected for over about 50 years from the survivors of the epidemic, and the most recent records refer to older Hitnü who knew the facts from the oral tradition of their parents. Results show that the Hitnü recognize the characteristics of a measles epidemic (“romé”). Moreover, symbolic questions followed explanations about the epidemic, and this study argues that their interpretations suffered the influence of the historical and sociopolitical context of the time, characterized by struggles between Indigenous and non-Indigenous people. This experience shows the value of Ethnography to reconstruct facts related to epidemics and explore health emergencies based on the perspective of the affected people to be reported by ethnographers who distance themselves from the often hegemonic narratives that reinforce inequalities, delegitimizing or ignoring the knowledge and resolution capacities of Indigenous people regarding this type of event and their interpretations, which, as with Hitnü and other South American groups, are linked to their relationship with non-Indigenous people over time.

## Abstract (pt)

Este artigo descreve a epidemia de sarampo de 1964 entre o povo Hitnü de Arauca, Colômbia, a partir da perspectiva dos próprios povos indígenas. Para tanto, foi realizado um estudo de Antropologia Histórica utilizando diários de campo de duas abordagens etnográficas (1976-1982 e 2023-2024). Os dados coletados durante aproximadamente 50 anos provêm dos sobreviventes da epidemia, e os registros mais recentes são de indígenas Hitnü mais velhos, que conheciam os fatos pela tradição oral de seus pais. Os resultados mostram que o povo Hitnü reconhece as características do que constitui uma epidemia (“romé”) de sarampo. Além disso, são levantadas questões de natureza simbólica que acompanham as explicações sobre a epidemia, e argumenta-se que suas interpretações foram influenciadas pelo contexto histórico e sociopolítico da época, caracterizado por lutas entre indígenas e não indígenas. Essa experiência mostra o valor da Etnografia para reconstruir fatos relacionados a epidemias e explorar emergências de saúde na perspectiva das pessoas afetadas e relatadas pelos etnógrafos, distanciados das frequentes narrativas hegemônicas, que reforçam desigualdades, deslegitimando ou ignorando não apenas o conhecimento e as capacidades de resolução dos indígenas em relação a esse tipo de evento, mas também suas interpretações, que como no caso dos Hitnü e de outros grupos da América do Sul estão vinculadas à relação que estabeleceram com os não indígenas ao longo do tempo.

## Introducción

Las epidemias son eventos masivos de enfermedad que ponen de manifiesto, de manera más evidente, la compleja relación entre la salud poblacional y los diversos procesos que suceden en una sociedad. Las epidemias acompañan a los humanos, cada vez de manera más frecuente, desde que empezó la agricultura y aparecieron los asentamientos con mayor concentración de personas, haciendo más común la convivencia con animales domésticos ^1^. Son tan importantes las epidemias en la historia que autores como Jörg Vögele, Luisa Rittershaus & Katharina Schuler señalan que las de mayor mortalidad pueden marcar un antes y un después de una sociedad ^2^.

La historia de las epidemias entre indígenas americanos durante la época de conquista europea, caracterizada por el intercambio sociocultural y de microorganismos entre poblaciones de ambos continentes ^3^, ha sido de especial interés entre los investigadores de epidemias. Los abordajes predominantes han sido las aproximaciones desde la demografía histórica, y la historia de la enfermedad fundamentadas en los datos y registros de los expertos de la época, muchas veces sin contar con las voces de los afectados. Entre los estudiosos de la historia de las epidemias que afectaron a los aborígenes americanos se destacan Alfred Crosby ^4^, Noble David Cook ^3^ y Massimo Livi Bacci ^5^, entre otros.

En este contexto, este trabajo describe, desde la perspectiva de los indígenas Hitnü, la primera epidemia de sarampión ocurrida en 1964. Se buscó tener una comprensión de las particularidades culturales de los Hitnü, lo cual suele ser difícil debido a las desigualdades étnicas que posicionan, tanto social como económicamente, a los grupos indígenas en los niveles inferiores de la sociedad. Este trabajo innova en la forma en que se estudian históricamente las epidemias, usando datos etnográficos de diferentes periodos de tiempo, que recuperan las vivencias de los afectados y sus descendientes.

## Material y métodos

### Contexto

**Figura 1:**
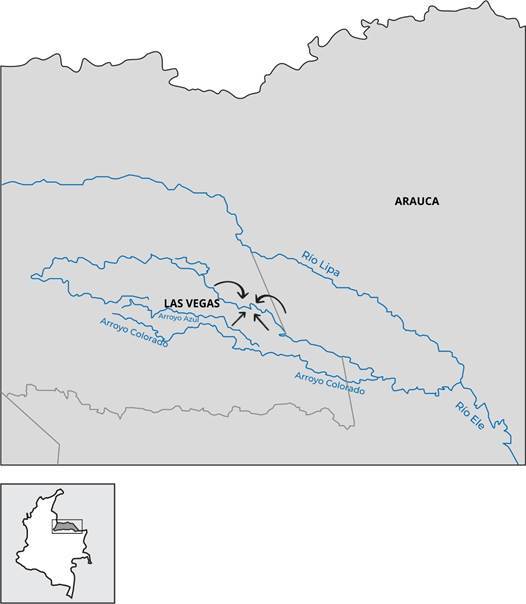
Localización del territorio Hitnü en el momento de la epidemia de sarampión. Arauca, Colombia.

El pueblo Hitnü es un grupo indígena de cazadores-recolectores. Para finales de 2024 eran aproximadamente 650 individuos, los cuales, desde 2004, fueron declarados en riesgo de desaparición por la Corte Constitucional de Colombia. El territorio ancestral de este pueblo ha sido la selva de Arauca (Colombia), también conocida como la gran selva del Macaguane, Airico de Macaguane o selva del Sarare ^6^, donde el río Arauca sirve de frontera con Venezuela (Figura 1). La riqueza de esta selva está en su potencial agrícola, ganadero y petrolero, lo que ha generado disputas entre diferentes actores y ha marcado la violencia durante décadas en esta zona. Hasta hace 70 años aproximadamente, los Hitnü obtenían grandes beneficios del territorio, al poder desplazarse de manera libre para realizar actividades de siembra, recolección, caza y pesca ^6^. Sin embargo, con el paso del tiempo, el pueblo Hitnü tuvo la necesidad de defender sus tierras para el logro de su supervivencia física y cultural ^6^.

Las primeras incursiones de foráneos en Arauca fueron en 1534, con la llegada de un grupo de alemanes. Posteriormente, arribaron las misiones jesuitas en 1628 y las misiones capuchinas en 1789, las cuales buscaban la evangelización de los indígenas. No se conoce de contactos entre los Hitnü y foráneos en este periodo. Los primeros colonos llegaron hacia 1930, quienes entre 1950 y 1960 participaban de las denominadas “guahibadas”, o cacería de indios ^6^ ^,^ ^7^; hay relatos que indican que algunos Hitnü fueron asesinados en este periodo. A finales de los años 1950 y la década de los 1960 inició la explotación petrolera, apareció la guerrilla del Ejército de Liberación Nacional (ELN), y colonos motivados por la colonización agrícola ^8^. Con la llegada de los colonos, los Hitnü empezaron a perder territorio, reduciéndose a un tercio del área original adjudicada por el estado en 1972 ^9^.

Pese a estos primeros contactos, hasta la década de 1980, los Hitnü eran los indígenas de Arauca con menos contacto con el mundo occidental ^7^. Aunque la Iglesia Católica tuvo presencia en la región durante los siglos XVII y XVIII a través de las misiones jesuitas y capuchinas, la selva protegió a los indígenas del influjo católico ^10^. Para los años 1970, las gripes y diarreas, dermatosis, enfermedad de Chagas, tuberculosis y desnutrición, se atribuían al contacto con foráneos ^11^. Estas enfermedades aún se observan entre los Hitnü ^12^. La violencia en su territorio, ha generado el desplazamiento masivo de parte de su comunidad a la ciudad de Arauca, obligándolos a una rápida transición del nomadismo al sedentarismo, sin seguridad alimentaria y grandes cambios en su estilo de vida.

### Tipo de estudio

Se realizó un estudio de Antropología Histórica ^13^, o reconstrucción histórica basada en las voces de sus protagonistas, registradas con etnografía. Se usaron dos tipos de registros etnográficos; unos fueron recolectados directamente, hace casi 50 años, de los sobrevivientes de la epidemia, y los otros registros fueron obtenidos a finales de 2023 y comienzos de 2024 de indígenas Hitnü mayores, que conocieron los hechos por tradición oral de sus padres. Esto permitió contrastar y complementar los relatos.

La primera fuente correspondió a un conjunto de diarios de campo escritos por Miguel Lobo-Guerrero, quien tuvo contacto con los Hitnü entre 1976 y 1982. Estos corresponden a ocho cuadernos (Cuadro 1) facilitados por la Fundación Etnollano. En ellos, Lobo-Guerrero narra lo que observó y plasma sus impresiones mientras convivió con este pueblo, explorando sobre diferentes acontecimientos de su cotidianidad, entre ellos la epidemia. Los registros etnográficos de 2023-2024 fueron recolectados por la autora principal (C. Amaya-Castellanos), en un trabajo realizado con un grupo Hitnü de 67 miembros de la comunidad El Alcaraván, desplazados por la violencia, que vive en la zona urbana de la ciudad de Arauca. En diferentes momentos Amaya-Castellanos, asumiendo un rol de profesora que enseñaba a leer y escribir el español, leyó a los mayores los registros etnográficos de Lobo-Guerrero sobre la epidemia. Allí, miembros de la comunidad, incluido el cacique y el profesor, comentaron sobre el suceso, complementaron, corrigieron y/o reafirmaron los pormenores de dicho evento. Algunos de estos relatos fueron grabados, y todos registrados en un diario de campo. El estudio fue explicado verbalmente y el consentimiento fue leído y explicado siguiendo sus costumbres. Para manejar el anonimato, se colocaron nombres ficticios de personas y familias. El estudio fue aprobado por el comité de ética de la Universidad Rovira i Virgili (España; código CEIPSA-2023-TD-0065).

**Cuadro 1:** Contenido de los cuadernos de campo de la etnografía realizada por Miguel Lobo-Guerrero.

CUADERNO	FECHA	CONTENIDO
#1 y #2	21 de Diciembre de 1975 a 31 de Enero 31 de 1976 / Mayo a Julio de 1976	Descripción de la reserva indígena; relatos sobre las cacerías indígenas, las vivencias durante el verano y el invierno, las plantas y la cura, la alimentación, el parentesco y ciclo de vida
#3	10 de Noviembre de 1976 a 30 de Enero de 1977 / Julio a Agosto de 1977	Descripción de la cacería, la pesca y la búsqueda de miel. Relatos sobre el yopo y el tabaco, algunas prácticas curativas y ceremonias; dibujos de las fuentes hídricas, casas y familiogramas
#5 y #6	9 a 16 de Noviembre de 1978 / 14 a 23 de Julio (sin año)	Relatos sobre muertes y desapariciones de indígenas por acciones de los blancos; historias sobre el yopo, el vino de palma y la brujería. Descripción de algunas patologías, del embarazo y el parto, y comentarios sobre las características de la medicina Hitnü
#7	Mayo a Junio de 1982	Etnomedicina Hitnü, especialistas, fisiología, anatomía, patologías, anomalías hereditarias, accidentes, ciclo de vida (menstruación, embarazo, parto, post parto y muerte) y algo de cosmología. Epidemia de sarampión
#8	Sin año exacto. Se presume 1981-1982	Descripción de enfermedades (causas, síntomas y tratamiento), denominación de términos de salud, datos sobre morbimortalidad y respuesta social organizada, sobre cosmovisión y ciclo vital. Epidemia de sarampión

El análisis de los datos se realizó a partir de categorías temáticas ^14^. Se identificaron y clasificaron en cinco temas relacionados con la epidemia de sarampión ocurrida en 1964: (1) evidencia de la ocurrencia de la epidemia: búsqueda de narrativas que validaran la existencia del evento, complementándolo con la evidencia de fuentes secundarias publicadas por Lobo-Guerrero u otros; (2) descripción de la enfermedad: denominación de la enfermedad por parte de los Hitnü y descripción de la misma y de aquellas características relacionadas con la sintomatología; (3) cosmovisión Hitnü de la epidemia: interpretación de los Hitnü sobre la aparición de la enfermedad; (4) impacto poblacional: identificación de datos en los diarios de campo, que pudieran dar muestra de los efectos demográficos a causa de la epidemia; y (5) manejo de la epidemia: acciones de los Hitnü para el control de la enfermedad.

## Resultados

### Evidencia de la epidemia

Algunos Hitnü relatan que hacia 1962, Gilberto Fernández, un colono ganadero logró convencer a buena parte de los indígenas Hitnü que habitaban en las riberas del río Ele y de los caños Colorado, Ele y El Dorado, para que se fueran a vivir cerca de su hato ^6^; otros indígenas le contaron a Lobo-Guerrero, que ellos sabían que había blancos buenos, que querían a los Hitnü y por eso lo habían buscado. Solo había cuatro casas de blancos, una de Gilberto Fernández, quien les dijo que se quedaran a vivir al lado de su casa. Él les hizo un pueblo, construyéndoles casas y una cancha para jugar pelota ^7^. Esto coincide con la versión de colonos de la época, que indican que los indígenas pidieron ayuda a Fernández, quien por su espíritu benefactor les cedió parte de su finca ^7^. Esta descripción sobre la bondad de Fernández ha permanecido. El actual cacique Hitnü refiere que “*este racional era un buen hombre y lo que hizo fue llevarse a todos los Hitnü. Les dio casa y les dio ropa, porque mis parientes usaban guayucos y las mujeres tenían los pechos descubiertos y eso no estaba bien, por lo que fue muy importante lo que hizo ese blanco por mi pueblo*” (Amaya-Castellanos, cuaderno de campo, día #7, 2023).

Así, los indígenas pasaron de habitar un territorio selvático a una zona de sabana y, dejaron de vivir en asentamientos dispersos, para establecerse en un único espacio más reducido; alrededor de 300 personas con patrones de seminomadismo se instalaron, cambiando radicalmente su forma de vida. En estas condiciones ocurrió la epidemia de sarampión en 1964, dos años después de haberse asentado en el lugar, lo que ocasionó la muerte de al menos 100 Hitnü ^6^.

Este hecho fue devastador para los indígenas sobrevivientes, quienes decidieron abandonar el lugar. En las notas se lee que “*después de que llevábamos algún tiempo en el pueblo, nos enfermamos todos. Romé (sarampión) mató a muchos de nosotros. Por eso ahora somos tan poquitos. Por eso no hay ahora gente vieja. Luego quemamos todos el pueblo y nos fuimos otra vez todos al monte*” ^7^ (p. 16).

Esta historia, al ser discutida con los Hitnü, ellos mismos aclaran que el concepto de *romé* hace referencia a una epidemia de enfermedad infecciosa, sin necesariamente restringirse al sarampión. Después de este evento, hasta 1982, cuando se realizó el trabajo etnográfico, no volvieron a ocurrir casos de la enfermedad, y las enfermedades respiratorias fueron catalogadas como *buk* (gripe), mucho más leve en su cuadro clínico.

### Descripción del sarampión

En las notas de campo, Lobo-Guerrero escribe que la palabra *romé* (epidemia), está relacionada con un ser mitológico denominado Roménu. La descripción de lo que significa “ocurrencia masiva”, es explicado por los Hitnü actuales, quienes señalan que “*romé, son como varias personas o niños que se afectan mucho. Es como el ganado, que son varias reses*” (Amaya-Castellanos, cuaderno de campo, día #63, 2024).

Roménu es descrito como un Hitnü joven a quien Nakuanu Tsetséri, creador de la tierra, convenció para que comiera gente; por eso se volvió malo. Lo describen como un “ladrón” u “hombre blanco” de baja estatura, frente grande, dientes largos y afilados, dedos grandes, cuerpo negro y cubierto de pelo, que “le rompe el corazón” (a la persona), ocasionándole la muerte. El adelgazamiento experimentado en la enfermedad se explica como el resultado de la grasa o manteca que Roménu saca de la persona, para comérsela con su esposa e hijos. Esto lo hace raspando con un tipo de cucharilla afilada, la cual puede cortar el corazón y matarlo, siendo el peor desenlace del *romé* (Lobo-Guerrero, cuaderno de campo #8, 1981).

Algunos sobrevivientes recuerdan que la enfermedad, además de producir el adelgazamiento del cuerpo, generaba brote en la piel (exantema), falta de apetito, vómito al comer, ojos rojos, dolor en los arcos de los ojos y estómago, malestar y pesadez en la cabeza (síntoma de que se va a morir pronto), sangrado por el ano y genitales (estado avanzado del sarampión), pérdida de la audición, debilidad generalizada, fiebre durante aproximadamente 10 días, y la ocurrencia de abortos entre las embarazadas (Lobo-Guerrero, cuadernos de campo #8, 1981, y #7 − Medicina Hitnü, 1982). Algo importante que Lobo-Guerrero destaca en sus notas es que si alguien sobrevivía con fiebre durante más de 10 días se asociaba con la recuperación (Lobo-Guerrero, cuaderno de campo #7 − Medicina Hitnü, 1982). Como característica adicional, los Hitnü actuales señalan la presencia de dolor en la pantorrilla y malestar en todo el cuerpo (Amaya-Castellanos, cuaderno de campo, día #63, 2024).

### Cosmovisión Hitnü de la epidemia

Según las narraciones, la epidemia de sarampión fue consecuencia de la muerte de Akír, un *mítsenü* (chamán o brujo experimentado) muy prestigioso, quien era un anciano cuando un grupo de blancos lo mató. Lobo-Guerrero relata que la historia sobre la muerte de Akír la conoció de un grupo de hombres Hitnü que estuvieron consumiendo *yopo* y mascando *yagé*, mientras estaban en un estado eufórico. El tema generó tristeza en los semblantes de quienes estaban conversando. Uno de ellos contó que Akír sabía mucho, tocaba la maraca y sabía cantar y soplar a los enfermos con *yopo* y tabaco. Que era un verdadero chamán, que había ido a vivir a ese lugar, buscando mujer. Que un día, a las 4 de la mañana, cuando aún era oscuro, del monte que rodeaba la casa empezó a “llover plomo” (balacera). Akír que estaba acurrucado tocando la maraca y cantando, cayó fulminado con los primeros tiros que le destrozaron la cara y el pecho. Ante el pánico, todos salieron corriendo hacia el monte, olvidando los arcos y las flechas. Desde el monte pudieron ver que era un grupo de 5 o 6 blancos armados con báculas (rifles de un solo tiro) y fusiles, que entraron a la casa y remataron a Akír a balazos. Le sacaron el corazón con un cuchillo a un niño que estaba llorando y gateando, y luego le prendieron candela a la casa con chinchorros, hamacas, arcos y flechas. Todos tuvieron que quedarse en el monte y dormir en el suelo, sin armas, sin casa y sin nada. Dicen que todos saben quién dirigía el grupo de blancos, que nunca pagó por ese crimen, pero que luego estuvo seis meses en la cárcel de Villavicencio por haber matado al dueño de un hato (Lobo-Guerrero, cuaderno de campo #5, 1978).

Relatos registrados en otro diario de campo, señalan que Akír se encontraba viviendo en la selva, mientras un número importante de indígenas Hitnü ya se había desplazado a vivir al nuevo poblado, cerca de Gilberto Fernández (Lobo-Guerrero, cuaderno de campo #8, 1981). Después de los relatos, Lobo-Guerrero dejó por escrito que para este pueblo la muerte de cualquier chamán acreditado genera desequilibrios en la naturaleza y la sociedad, afectando la vida a nivel grupal y familiar (Lobo-Guerrero, cuaderno de campo #8, 1981).

Además de este evento, Lobo-Guerrero narra otro suceso que explica la aparición de la epidemia, aunque menos detallado que la de Akír. Según los Hitnü “*fue la envidia de Inacho la que mato a toda la gente*”. Inacho era un Hitnü a quien los colonos le habían hecho regalos llamativos, como hachas, ollas, telas, azúcar, cigarrillos, antes que a los demás Hitnü, los cuales, al enterarse, bajaron de la selva para obtener estos regalos, lo que no agradó a Inacho. Por eso, estando en la nueva tierra cerca de Gilberto Fernández “*a todos les dio* [la enfermedad] *y solo la mitad de la gente se salvó*”. Aunque, dentro de quienes murieron, también hubo gente de Inacho, por la acción de Akír (Lobo-Guerrero, cuaderno de campo #8, 1981). El relato de este evento no se profundiza en ningún otro diario de campo, aunque respecto a la primera historia, Akír vuelve a aparecer como un personaje importante.

En los relatos actuales, el profesor de la comunidad, quien es Hitnü, se refiere a las dos versiones, la de Akír y la de Inacho, como causantes de la epidemia. Lo sabe por su papá adoptivo, quien, sin ser Hitnü, era muy cercano a ellos en la época de la epidemia. Dice que el apellido de Akír era Reyes, quien efectivamente era un chamán muy prestigioso, el cual con su poder generó la enfermedad cuando los blancos lo mataron. Aclara que cuando Akír genera la enfermedad, aparece Roménu. Respecto a Inacho, dice que este era un jefe igualito a Akír, otro jefe mayor que, por envidia, *chispeó* la enfermedad, es decir, rezó la enfermedad, lo que mató a muchos. Su mala acción hizo que no se le pudiera tocar, ni tampoco sus cosas, ni mirarlo a los ojos. Inacho a diferencia de Akír murió de vejez (Amaya-Castellanos, cuaderno de campo, día #17, 2023; día #74, 2024).

Existe un canto a Roménu, traducido por Lobo-Guerrero, en el que se acusa a Roménu de ser malvado, poderoso y de haber causado la enfermedad y muerte de muchos Hitnü, comiendo la carne, cortando y sacando la grasa de sus corazones (Cuadro 2). Roménu enferma a las personas a distancia, sin que nadie pueda verlo; solo lo ven los *mítsenü* experimentados, o cuando lo sacan del cuerpo de un enfermo en forma de una espina larga (Lobo-Guerrero, cuaderno de campo #8, 1981). Llama la atención que, para el cacique actual Hitnü, Roménu no es un ser tan malvado. Dice que tanto en el momento de la epidemia, como ahora, él solo se encarga de recoger a todas las personas y prepararlos para llevarlos al mundo de abajo, al mundo de los muertos (C. Amaya-Castellanos, cuaderno de campo, día #7, 2023).

**Cuadro 2:** Canto del Roménu del pueblo Hitnü.

*Oh Roménʉ, Roménʉ* *Tú comes nuestra carne* *Tú comes mi propia carne* *Tú mataste a toda mi gente* *Tú comiste la carne de mi gente* *Roménʉ, Roménʉ*
*Eres un Nakuánʉ malvado y poderoso* *Nos enfermaste y nos mataste* *Roménʉ, tú eres malo* *Con un cuchillo afilado, Roménʉ, Roménʉ, a todos nos fuiste cortando por dentro* *Poco a poco raspaste sus corazones* *Cortando y sacando la grasa de sus corazones* *Raspando la carne y grasa sin que murieran*
*Nakuánʉ Roménʉ está hambriento* *Así enfermaste a todos de la cabeza y oscureciste la fuerza de sus piernas* *A todos los enfermaste, a todos*
*Frío, nuestros corazones tienen frío* *Todos los muchachos se acabaron* *Se fueron muriendo y las casas fueron quedando solas* *Todos se fueron acabando* *Se fueron muriendo*
*Nakuánʉ Roménʉ* *Roménʉ, tú comiste la carne de mi gente* *Mi gente está enferma toda* *Eres bravo, eres malo* *Quédate abajo en tu tierra* *Tú ya comiste a mi gente* *Quédate en tu tierra* *A toda mi gente la enfermaste*

Fuente: traducción libre realizada por Miguel Lobo-Guerrero. Se han quitado las repeticiones de palabras y de frases que acompañan la melodía, pero el texto, propiamente dicho, permanece igual.

### Impacto de la epidemia

Para tener una aproximación al impacto que tuvo la epidemia, se consideraron datos demográficos posteriores, y los discursos de los sobrevivientes y notas registradas en los diarios de campo por Lobo-Guerrero. Cabe señalar que en los manuscritos se explicita que no existen registros de defunción que permitan dar con exactitud el número de fallecidos. Mucha de esta información fue brindada por los mismos Hitnü, quienes reportaron parientes muertos, y por la información de colonos y médicos que estuvieron al tanto del caso en esa época ^6^. Actualmente, los Hitnü recuerdan la epidemia como un evento triste que dejó mucha gente muerta, sin hablar de datos precisos de los afectados.

**Figura 2:**
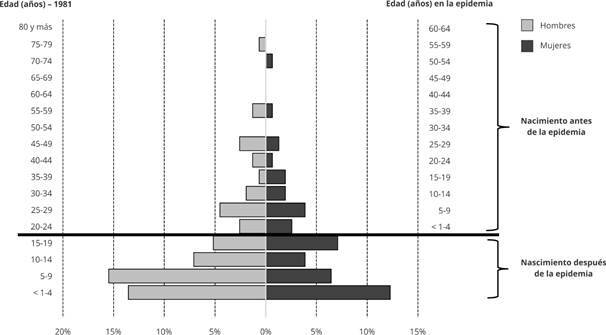
Pirámide poblacional del pueblo Hitnü mostrando el impacto de la epidemia de sarampión.

El registro etnográfico incluyó una descripción del pueblo Hitnü en relación con el sexo y la edad en 1981, 17 años después de ocurrida la epidemia. Con estos datos se construyó la pirámide poblacional (Figura 2), en la que se observa el fuerte impacto demográfico que tuvo la elevada mortalidad de la epidemia. Como se puede observar, la pirámide poblacional es bastante reducida entre los mayores de 20 años, quienes correspondían a los más pequeños en el momento de la epidemia. Por debajo de esta edad, se observa un número mucho mayor de individuos, lo que muestra el crecimiento poblacional posterior a la epidemia.

### Manejo de la epidemia

Los Hitnü en sus narrativas señalan que una enfermedad como la de *romé* (epidemia) solo podía ser manejada por un *mítsenü* que soplara y chupara los enfermos, hasta sacar del cuerpo a Roménu; esto se realizaba mientras se entonaba la canción a Roménu, descrita previamente. El éxito se observaba al sacar a Roménu en forma de espina larga, que era tirada lejos de la casa, logrando un poco de alivio en el enfermo (Lobo-Guerrero, cuaderno de campo #8, 1981). Sin embargo, parece que no había un *mítsenü* en el lugar donde se desató la epidemia, por lo que no se pudieron realizar las acciones para manejar la enfermedad. No debe olvidarse que Akír había sido asesinado, quien pudiera ser el indicado para manejar la epidemia. Este papel del *mítsenü* como el único con la capacidad de manejar la enfermedad, es reafirmado por los Hitnü en la actualidad, quienes señalan que debió ser muy difícil controlar la epidemia sin un *mítsenü* o cacique, quienes son los únicos con el poder para decidir qué hacer ante una enfermedad, que comer o no, cuando tomar agua, cuando salir al sol, o cuando ir a trabajar. Si se contravienen sus órdenes, la persona puede morir y el *mítsenü* o cacique pierden poder (C. Amaya-Castellanos, cuaderno de campo, día #63, 2024). El tomar la decisión de abandonar los lugares de vivienda y dispersarse fue la forma en que se manejó, de manera intuitiva, la epidemia.

## Discusión

Este trabajo evidencia la epidemia de sarampión de 1964 ocurrida entre el pueblo Hitnü, siendo el evento más trascendental en su historia por el número de muertes ocasionado. La reconstrucción de los hechos se fundamentó en las voces de sobrevivientes y descendientes de estos, logrado mediante etnografía. Contrario a la aproximación de los expertos a situaciones similares, que suelen asociar una epidemia con el hacinamiento y la vivencia conjunta de varios individuos en un lugar de tamaño reducido, para los Hitnü su ocurrencia se relaciona con la muerte de una figura prestigiosa como lo representa un mitsenú y la envidia de un Hitnü, igualmente acreditado por su comunidad.

Específicamente, sus explicaciones sobre el origen del sarampión coinciden con las narrativas orales de aproximaciones etnográficas sobre el origen de las epidemias y de las enfermedades graves en general, en las cuales la etiología se atribuye a fuerzas poderosas resultado del enfrentamiento entre actores significativos, que afectan la cotidianidad ^15^, o entre seres poderosos y predadores ^16^, como lo representa Roménu para el pueblo Hitnü. Estas interpretaciones también mantienen relación con las de la mayoría de los pueblos indígenas de América del Sur, que atribuyen las enfermedades al contacto con población no indígena, a espíritus, demonios o dioses malévolos, al igual que a la brujería humana, la cual puede ser causada, por los mismos miembros de la comunidad o por grupos indígenas o no indígenas ^17^. Sus manifestaciones clínicas igualmente envuelven elementos simbólicos, como en los Hitnü, quienes explican el adelgazamiento que produce el sarampión, como la grasa o manteca que Roménu saca de la persona para comérsela. O como en el caso de los Desana que habitan el alto río Negro en Brasil, que relacionan los exantemas propios de la enfermedad con cuencas de vidrio, las cuales suelen utilizar como objetos que usan como divisas de intercambio con los pueblos no indígenas ^17^.

Si bien el componente simbólico parece mostrarse como un elemento preponderante en las explicaciones sobre la enfermedad, este responde a un contexto que no puede obviarse. Entre los Hitnü las versiones de la muerte de Akír y la envidia de Inacho, como detonantes para la aparición del *romé*, reflejan las relaciones existentes entre blancos e indígenas en ese momento. Frente a las narrativas sobre Akír, se evidencian las tensiones existentes con los blancos, siendo vistos como amenaza, mientras que en el caso de Inacho, se identifica al blanco como persona buena, que brindó beneficios a algunos indígenas. Este tipo de interpretaciones también se dieron entre los Barí de Venezuela, quienes atribuyeron su primera epidemia de sarampión (1960-1962), al aterrizaje de un helicóptero con algunos hombres blancos (misioneros) que les brindaron galletas y una bebida roja, con lo que, según ellos, fueron envenenados, generando la muerte de muchos en pocas horas. Algunos sobrevivientes relatan que se salvaron al recordar las intenciones de blancos en el pasado, quienes les ponían sal envenenada, al saber que les gustaba comerla ^18^. Este tipo de narrativas también están presentes entre indígenas de la Amazonía de Brasil; sus interpretaciones, sobre algunas enfermedades infecciosas, entre ellas el sarampión, han sido influenciadas por el contexto histórico y sociopolítico del momento, asociándolas al contacto con las sociedades no indígenas y diferenciándolas de sus enfermedades propias, por su eventualidad y contagio ^17^.

En síntesis, todas estas narrativas, ponen de manifiesto las relaciones sociales establecidas entre indígenas y no indígenas, identificados como criollos, hacendados o misioneros, en su mayoría denominados “blancos”. Estas relaciones parecían caracterizarse, salvo por algunos blancos bondadosos, por la desconfianza, el resentimiento y la tristeza, sentimiento último que Lobo-Guerrero describe frente al relato que hace un Hitnü sobre la muerte de Akír. Es claro, entonces, que en todos estos casos la acción de agentes externos como generador de la enfermedad es un elemento común de las epidemias ^19^, y que la interpretación de todos los elementos simbólicos está sujeta a un determinante de tipo social ^20^.

Respecto a las acciones que los indígenas toman ante las epidemias, estas están ligadas a su cultura, como sistema simbólico que les permite interpretar el mundo y actuar ^21^. Por ejemplo, de los Yanomami se sabe que cuando uno de sus jefes enfermó de sarampión, varios miembros de su pueblo lo visitaron. Luego fueron con otros, contando las cosas tristes y de desesperanza que habían visto. Esta costumbre de reunirse fue clave para la transmisión de la epidemia de sarampión entre este grupo ^22^. En el caso de los Hitnü, sus acciones, una vez ocurrida la epidemia, coinciden con las prácticas de aislamiento realizadas por otros grupos indígenas, como entre los Barí, quienes se presume que, por miedo a la epidemia de sarampión, abandonaron y quemaron sus casas, aislándose temporalmente en las montañas ^18^. En otros grupos, incluso fuera de América, además del aislamiento, se dio el abandono de los enfermos para evitar la propagación ^23^.

Esta historia del pueblo Hitnü tiene un contexto compartido con muchos aborígenes suramericanos. Después de los periodos de Conquista del Nuevo Mundo, iniciados a finales del siglo XV, este tipo de historias se han repetido entre indígenas de la Amazonía, que se mantuvieron aislados de los no indígenas. Entre las historias más conocidas son las epidemias de viruela, sarampión, tos ferina, gripe y “fiebres intermitentes” entre el pueblo Desana del río Negro; los estudios etnográficos muestran que, desde los primeros contactos con portugueses en 1730, las epidemias se repitieron hasta el siglo XX con efectos demográficos desastrosos ^24^. También se recuerdan las epidemias que llevaron a la muerte de gran parte del pueblo Yanomami del sur venezolano y norte brasilero. Este caso se usó como ejemplo en un gran debate entre antropólogos sobre el rol que las enfermedades infecciosas tuvieron en la disminución abrupta de poblaciones sin inmunidad ^25^. De allí la importancia de escuchar las voces de los propios indígenas que sobrevivieron a las epidemias.

También se destaca el trabajo clásico de Darcy Ribeiro, quien describió diversas epidemias en el Amazonas. Sobresalen las epidemias de gripe entre los Kaingang, Xokleng y Urubu (1912 y 1913); la de sarampión entre los Xokleng (1927); las de tuberculosis entre los Karajá, de gripe entre los Tucano y de sarampión entre los Urubu, todas estas hacia 1950; la epidemia de varicela entre los Bororo (1953); las de sarampión entre los Tuparí, Kakurap, Arikapu, Xingu y Jabutí (1954), y la sucesión de epidemias entre los Xokleng: malaria (1917), coqueluche (1918), gripe española (1919), sarampión y parotiditis (1927), y gonorrea (1939) ^26^.

Otros trabajos en Brasil han reportado epidemias entre indígenas del Araguaia medio, como los Karajá, Javaé y Tapirapé, que llevaron a su hecatombe durante la primera mitad del siglo XX ^27^, y muchos pueblos al norte del río Amazonas. Entre estos sobresalen los pueblos Makuxi, Waiapi, Waimiri-atroari Waiwai, Wapishana y Wayana-aparai ^28^. En otros países amazónicos, los trabajos etnográficos también han descrito epidemias. Durante las primeras tres décadas del siglo XX, el pueblo Siona del río Putumayo, en el suroccidente colombiano, disminuyó su población en un 75%, principalmente, por epidemias de sarampión, tos ferina e influenza ^29^. Recientemente, en el contexto de la pandemia de COVID-19, Espinosa & Fabiano ^30^ lideraron una compilación de experiencias de pueblos amazónicos peruanos durante epidemias. Así, los descendientes de los sobrevivientes de epidemias de viruela, sarampión y gripe, pudieron relatar como los caucheros, colonos, misioneros, madereros y mineros llevaron la enfermedad a la selva, ocasionando que muchos pueblos huyeran y se aislaran, como medidas de protección frente a las epidemias.

Estas experiencias entre los indígenas suramericanos muestran el valor de la Etnografía, la cual ha permitido reconstruir hechos relacionados con epidemias. Las narrativas de estos grupos frente al evento del sarampión, desde su propia perspectiva y relatadas por etnógrafos, han permitido ver a los sujetos que viven epidemias más allá de los eventos masivos y cuerpos fijos que experimentan una enfermedad, como cuerpos sociales ^31^ que interpretan su realidad, dotando de significado los eventos que emergen en su contexto. Esta capacidad de producir y transmitir significados, además, corresponde a un momento histórico y a una sociedad particular ^18^. Es por ello que las aproximaciones etnográficas explican las epidemias como crisis de tipo biopsicosocial, generadas por factores políticos, económicos y socioculturales, que terminan produciendo violencia y desigualdad en las poblaciones que las experimentan ^32^.

Más recientemente, la importancia de la Antropología, y del abordaje etnográfico en particular, en el estudio de las epidemias se ha visto fortalecida, no solo por su papel en la comprensión del origen social de estos eventos, sino en la posibilidad de explorar, desde otra perspectiva, las emergencias de tipo epidémico que no logran ser captadas por la biomedicina ^33^. Esta aproximación refuerza las desigualdades, deslegitimando o ignorando no solo los conocimientos y capacidades resolutivas de los indígenas respecto a este tipo de eventos, sino sus interpretaciones, las cuales como en el caso de los Hitnü y otros grupos del sur de América, están vinculados con el relacionamiento que han establecido con los no indígenas a través del tiempo.

En este sentido, son tan válidas las interpretaciones relacionadas con los virus para explicar el origen de la epidemia y su capacidad de transmisión y contagio por contacto, como aquellas, resultado de luchas o el egoísmo, como en el caso de los Hitnü. Incluso en ambas interpretaciones, las expresiones sobre el relacionamiento entre individuos se muestran como un elemento común, sin lo que no podría transmitirse la enfermedad. Esto muestra, cómo las cuestiones relacionadas con los procesos de salud-enfermedad-atención deben considerarse desde las perspectivas de los entornos socioculturales únicos en los que ocurren ^34^. La comprensión de estas particularidades se constituye en elemento central para el manejo efectivo de situaciones complejas como lo constituyen las epidemias.

En conclusión, este estudio de Antropología Histórica evidencia la ocurrencia de una epidemia de sarampión, en 1964, entre los Hitnü. Dado que los hechos ocurrieron apenas unos años después de tener los primeros contactos permanentes con los colonos, deben ser similares a lo sucedido en los primeros encuentros entre aborígenes y europeos durante el periodo de Conquista de América. Sin embargo, el método de la antropología histórica permitió no solo evidenciar la epidemia, sino entender cómo la percibieron y comprendieron los indígenas, desde sus propios saberes y cosmología. Sin duda, la pandemia de COVID-19 renovó la importancia de conocer la historia de las epidemias entre los pueblos aborígenes americanos, pues las similitudes observadas permiten ver que las epidemias son hechos que se repiten muchas veces, en diferentes lugares, afectando poblaciones vulnerables como los indígenas ^35^. Como bien ha sido señalado por Santos et al. ^36^, las epidemias son “hechos sociales totales” que evidencian las tensiones generadas por las políticas públicas dirigidas a indígenas; estas deberían ser comprendidas para lograr un mejor abordaje intercultural en futuras crisis sanitarias.
